# Preliminary survey of dengue, Zika and chikungunya viruses by nucleic acid test in blood donors in Shenzhen, China

**DOI:** 10.3389/fpubh.2025.1672814

**Published:** 2025-12-05

**Authors:** Xin Zheng, Xipeng Yan, Xiaoxuan Xu, Liu Heng, Xiujuan Cui, Linfeng Wu, Ran Li, Litao Wu, Limin Chen, Jingfeng Zeng

**Affiliations:** 1 Shenzhen Blood Center, Shenzhen, China; 2 The Joint Laboratory of Transfusion-Transmitted Diseases (TTDs) between Institute of Blood Transfusion, Chinese Academy of Medical Sciences, Peking Union Medical College, Nanning Blood Center, Nanning, China; 3 Institute of Blood Transfusion, Chinese Academy of Medical Sciences, Peking Union Medical College, Chengdu, China

**Keywords:** blood donation, denv, ZIKV, CHIKV, nucleic acid test, prevalence, blood safety

## Abstract

**Objective:**

To investigate the prevalence of dengue virus (DENV), Zika virus (ZIKV), and chikungunya virus (CHIKV) in blood donors and to preliminarily assess the potential risk of arbovirus transmission through blood transfusion in Shenzhen, China.

**Methods:**

The Nucleic Acid Fully Automatic Mixing Extraction Analysis System from China’s Kehua Biotechnology Company was used to screen for RNAs of DENV, ZIKV, and CHIKV in plasma samples from Mini pools of 8 voluntary blood donors (MP-8). The nucleic acid test results were summarized and analyzed.

**Results:**

7,520 (75.13%, 7520/10009) and 2,489 (24.87%, 2489/10009) voluntary blood donations were collected from Shenzhen Blood Center and Bao’an District Central Blood Station, respectively, from August 27 to October 18, 2022 for nucleic acid testing of DENV, ZIKV, and CHIKV. Eight donations were mixed to 1 mini-pool (MP-8) and 1,344 test reagents were used for detecting these 1,252 mini-pools. No reactivity was obtained from these 1,252 MP-8 donations. Our results indicated there was an extremely low acute infection rate of DENV, ZIKV, and CHIKV in the surveyed 10,009 blood donations during the summer season in Shenzhen although it is located in the subtropical region of southern China, where *Aedes albopictus* mosquitoes breed abundantly. This is consistent with previous reports that the positive rate of the nucleic acid testing for DENV, ZIKV, and CHIKV in blood donors is extremely low in China during non-arthropod-borne virus epidemics.

**Conclusion:**

The positive rate of acute infections by DENV, ZIKV, and CHIKV in blood donors in arbovirus non-endemic areas in southern China is extremely low, and it is suggested to perform nucleic acid screening only during outbreaks or epidemics. This study not only provides preliminary data on the prevalence and risk assessment of acute infections by DENV, ZIKV, and CHIKV in the blood donor population in Shenzhen, but also equips the technical reserve capacity for emergency screening during unexpected outbreaks of arbovirus epidemics in the region in order to ensure blood safety.

## 1 Introduction

Hepatitis B virus (HBV), hepatitis C virus (HCV), and human immunodeficiency virus (HIV), as routine pathogens mandatorily screened for blood donations, have an extremely low probability of transmission through transfusion (1). However, the emergence of new pathogens transmitted through blood transfusion poses new challenges to blood safety. The World Health Organization (WHO) recommends that blood centers with testing capabilities in various regions conduct epidemiological surveys on the prevalence of the emerging pathogens in blood donors and assess their risks to blood safety (2). Among these emerging pathogens, rapidly spreading arboviruses, such as dengue virus (DENV), Zika virus (ZIKV), and chikungunya virus (CHIKV) are of particular concern. These arboviruses pose threats to public health and transfusion safety, which attracts global attention due to their increasing global incidence and widespread vector distribution (3), and potential to be transmissible through transfusion (4). DENV, ZIKV, and CHIKV are primarily transmitted by mosquitoes, notably *Aedes aegypti* and *Aedes albopictus*, which are prevalent in tropical and subtropical regions, exposing large populations in these areas to infection (5). Shenzhen is located in Guangdong Province, China with a typical subtropical monsoon climate. Guangdong has been recognized as China’s highest-risk region for dengue transmission, with over 70,000 cumulative cases reported since 1990 (accounting for 69.7% of the national total cases during this period). As a major international metropolis with frequent population movements, Shenzhen faces a non-negligible risk for the introduction and spread of such vector-borne diseases (6). Because mandatory screening for ZIKV, CHIKV, and DENV (ZCD) is not performed in China, it is necessary to conduct a preliminary survey on the prevalence of ZCD in blood donors in this city. Results from this survey will add evidence for the policy-makers to decide whether adopting NAT screening for these mosquito-borne viruses in blood donors is necessary.

## 2 Materials and methods

### 2.1 Blood donors and blood samples

All blood donations were obtained from voluntary blood donors at Shenzhen Blood Center and Bao’an District Central Blood Station from August 27 to October 18, 2022. All blood donors provided informed consent for the use of their residual samples for scientific research. The leftover plasma samples from routine HBV, HCV, and HIV nucleic acid testing were used in this survey and were stored at 4 °C. The NAT screening for ZIKV, CHIKV and DENV was performed within 24 h post collection as required by the reagents manufacturer. This study was approved by the ethical committee of the Shenzhen Blood Center (Approval No: SZCMEC-REC-2021-011).

### 2.2 Reagents and equipment

Triplex viral nucleic acid reagent (Shanghai Kehua Company, China, Reagent Lot No. 20220705) for DENV, ZIKV, and CHIKV nucleic acid detection was used. The reagent demonstrates detection limits of 40 copies/mL for DENV, 44 copies/mL for ZIKV, and 25 copies/mL for CHIKV in individual testing (ID-NAT). For sample mixing, nucleic acid extraction and PCR, blood sample mixing and nucleic acid extraction system (STAR 645B, Hamilton, Switzerland) and PCR amplification instrument (Gentier 96R, Xi’an Tianlong Technology Co., Ltd., China) were used in this study.

### 2.3 Detection scheme

Screening for DENV, ZIKV, and CHIKV RNAs was conducted using the left-over blood samples after all HBV/HCV/HIV viral nucleic acid tests were completed. To prevent contamination, we adhered to standard operating procedures for routine nucleic acid testing. A pooled testing strategy was used. Briefly, eight samples were combined into one mini-pool (MP-8): 50 μL of plasma from each donation was pooled to create a 400 μL mixture for nucleic acid extraction and subsequent PCR amplification. Theoretically, reducing the extraction volume would compromise sensitivity.

## 3 Results

For quality control, we included both positive control for 3 viruses and negative control. As seen in Table 1, the performance is very stable and CV% is between 1.34 and 2.60% from 15 times repeated screening. In total, 1,344 test kits were used in this study, 77 (77/1,344) of which were used for pre-use calibration of the equipment system, 15 (15/1,344) were used for the positive and negative controls for each batch of testing, and 1,252 (1,252/1,344) were used for the MP-8 pool testing of blood specimens from blood donors. The effective utilization rate of the test kits was 93.15% (1,252/1,344). (Table 2).

**Table 1:** Quality control for nucleic acid detection of dengue, Zika and chikungunya viruses in 10,009 blood donations.

Positive control: FAM(DENV), Texas Red (ZIKV), CY5 (CHIKV)			
Program	Testing times	Average CT	CV%
HEX(IC)	15	26.34	1.34
FAM(DENV)	15	26.75	2.38
Texas red (ZIKV)	15	23.64	1.38
CY5(CHIKV)	15	25.8	2.08
Negative control: HEX(IC)			
Program	Testing times	Average CT	CV%
HEX(IC)	15	26.58	2.6

**Table 2:** Screening results and reagent availability of nucleic acid detection of dengue, Zika and chikungunya viruses in 10,009 blood donations.

Reagents (for 10,009 donations)				
Result	Donor’s samples	System adjusting	QC	Sample/reagent availability
Tests	1,252(10009)	77	15	1252/1344
Positive	0	-	-	-
Rate	0%	5.73%	1.12%	93.15%

The 1,252 test kits were used in an 8-in-1 pool testing mode to detect 10,009 blood specimens from blood donors. Of the 10,009 specimens, 7,520 were from blood donors in the urban areas of Shenzhen (collected by the Shenzhen Blood Center), and 2,489 were from blood donors in the suburban areas of Shenzhen (collected by the Bao’an Blood Center). The 1,252 pooled testing results were all negative, indicating that the prevalence of DENV, ZIKV, or CHIKV were extremely low in these 10,009 blood donor specimens. (Table 2).

## 4 Discussion

Between 1990 and 2019, a total of 92,995 local dengue fever cases were reported in China, with approximately 69.7% (75,350 cases) occurring in Guangdong Province, demonstrating an upward trend since 1990 (7, 8). Within Guangdong, the majority of cases (89.7%, 56,345 cases) were concentrated in cities of the Guangdong-Hong Kong-Macao Greater Bay Area, such as Guangzhou and Shenzhen (6, 9). Recent monitoring data indicated over 10,000 reported dengue cases in Guangdong during the September–October 2024 epidemic period. While the prevalence of ZIKV and CHIKV is lower in China, Guangdong remains a region of concern, with 7 imported ZIKV cases reported in 2016 and sporadic CHIKV outbreaks, including one in Yangjiang in 2010 (10–12). Notably, since the first imported case of chikungunya fever was reported in Foshan on July 8, 2025, a total of 9,933 cases have been reported. These recent arbovirus outbreaks underscore the potential threat to blood safety and justify the consideration of targeted nucleic acid testing (NAT) screening for blood donors during epidemic periods.

We performed this current preliminary survey in Shenzhen. The study period spans from August to October, corresponding to the local summer season and the peak epidemic period for arboviral diseases due to increased mosquito breeding.

In this study, nucleic acid screening of 10,009 blood donations using an MP-8 pooling strategy for DENV, ZIKV, and CHIKV yielded no positive results, indicating an extremely low prevalence of acute arboviral infections among donors in Shenzhen during a non-epidemic period. This finding aligns with a global meta-analysis, which reported low NAT positivity rates for these viruses in blood donors during inter-epidemic phases, often falling to 0%, even in endemic regions (13). The significantly higher rates observed during outbreaks underscore a tangible risk to blood safety (13–15). Consequently, implementing targeted NAT screening is justified during epidemic periods or in high-risk populations to mitigate transfusion-transmission risk. However, during non-epidemic intervals, routine NAT screening appears unnecessary, suggesting a more cost-effective, risk-based approach is feasible. In addition to NAT, serological screening of IgM and IgG antibodies of DENV, ZIKV and CHIKV signifies prior exposure to these viruses. As the testing cost is lower than that of NAT, it can be used in high-endemic countries for blood screening (16, 17).

Arboviruses pose a significant threat to the population, with a high incidence of asymptomatic cases. The presence of viruses in blood before the onset of symptoms was documented (18, 19). This makes blood-borne arboviruses more alarming because infected donors may transmit these viruses to blood recipients through transfusion. The study period for this project was not during an outbreak but in the hot summer season in Shenzhen, and summer is the breeding period for mosquitoes. Although no arboviral RNA was detected in this study, it does not mean that the infection rate of DENV, ZIKV, and CHIKV in the blood donor community in Shenzhen is zero. The absence of arbovirus outbreaks, the relatively small number of samples screened, and strict donor consultation before blood donation may account for our negative results from NAT screening.

Some limitations exist in our current study: In our pooled testing strategy, the sensitivity of the triplex detection reagent—determined to be 40, 44, and 25 copies/mL for DENV, ZIKV, and CHIKV, respectively, under ID-NAT (400 μL plasma input)—was compromised when employing an 8-sample pooling approach. With each sample contributing only 50 μL to the 400 μL mini-pool, the reduced input volume resulted in insufficient template quantity to achieve sensitivity levels equivalent to those of single-sample testing. The pooled testing approach, characterized by elevated detection limits, may fail to detect infections with low viral loads. However, when the infection prevalence is low, the pooling method is substantially more efficient than individual testing, so it is essential to optimize resource utilization while maintaining screening efficacy. In contemporary blood screening practice, the mini-pool testing strategy is predominantly adopted, which strategically compromises analytical sensitivity to achieve enhanced testing throughput and reduced operational costs, while maintaining assured blood safety.

## Data Availability

The original contributions presented in the study are included in the article/supplementary material, further inquiries can be directed to the corresponding author/s.
